# “Nomen Omen”: Exploring Connected Healthcare through the Perspective of Name Omen

**DOI:** 10.3390/healthcare8010066

**Published:** 2020-03-23

**Authors:** Sonia Chien-I Chen, Chenglian Liu, Ridong Hu, Yiyi Mo, Xiupin Ye

**Affiliations:** 1 Institute of Quantitative Economics, Huaqiao University, Xiamen 362021, Fujian, China; chien-c@hqu.edu.cn; 2 School of Computing, Neusoft Institute of Guangdong, Foshan 528225, China; chenglian.liu@gmail.com; 3 College of Civil engineering, Huaqiao University, Xiamen 361021, Fujian, China; myy_11@hotmaill.com (Y.M.); xiuyer@hqu.edu.cn (X.Y.)

**Keywords:** connected healthcare, aging society, health accessibility, e-health, telemedicine, telehealth, telecare

## Abstract

*Background*: The evolution of names, from “medical informatics” to “connected health”, implies that the evolvement of technology in health care has been shifted from technology-oriented to healthcare-oriented implementation. Connected healthcare, a healthcare platform of remote monitoring and self-management through technological measures, is suggested to contribute to the efficiency, cost-effectiveness, and satisfaction of healthcare recipient enhancement. However, limited understanding of related connected health (CH) terminology may constrain its implementation. Whether CH is a buzzword only or a practice that can contribute to an aging society is controversial. *Objective*: This study aims to distinguish CH-related terminology and to identify the trend of CH through reviewing its definition, initiation, development, and evolvement, in order to offer management insights and implications. The objective is to understand what is connected and who is cared about in the connected health model so that better applications can be addressed for the benefit of society. *Method*: This study reviews the evolution of names, from “medical informatics” in the 1970s to “connected health” after 2000, as well as relevant literature of CH, including e-health, telemedicine, telehealth, telecare, and m-health, to discover the trend of technology-related healthcare innovations. *Results*: The current status and issues facing accessibility, quality, and cost were presented. Its future trends will be explored through reviewing how changes in healthcare are managed, in addition to its operation and practice. Pre-conditions and requirements for implementing CH are identified to select a typical case to study. Findings suggest that areas with a complete business ecosystem—isolated locations, advanced information technology, aging in population, integrated health, and social care system—are prevalent for designing friendly CH environments. *Conclusion*: The evidence and tendency of technological convergence create a demand for innovation and partnering with start-up companies that offer a competitive advantage in innovation.

## 1. Introduction

Research into connected health (CH) is significant, as people all over the world are suffering from the challenges of an aging population and healthcare issues [1,2]. An initial review of CH shows that most research focuses on its performance in terms of cost-effectiveness and efficiency. This research further explores the relationship between technology and wellness and their optimization within the macro environment.

The rise of CH can be summarized as resulting from the following three factors. Firstly, there is a tendency to pursue excellence in healthcare, including via the promotion and monitoring of quality, efficiency, safety, and customer service [3]. Health professionals and institutions desire better access, quality, and efficiency of care [4,5,6,7]. Secondly, there are rapidly increasing costs caused by an aging population, an increase in chronic conditions, better survival rates among patients fighting serious diseases, and longer lifespans [8]. The healthcare economy has thus become more dynamic than in the past, as can be seen from rising costs and changing demographics [9]. Thirdly, increasing provider shortages and relevant issues, such as the geographic dispersion of families and troubling ethnic disparities in care, are significant concerns [10]. Finally, the latest source of pressure in healthcare is the demand for a better service by customers [11,12]. The development of consumerism in healthcare may be a catalyst for patient-centric healthcare [13]. These factors coming together have created a stronger impetus to force innovation both from within and outside the system.

In this study, the current status and issues facing accessibility, quality, and cost are presented. Its future trends are explored by reviewing how changes in healthcare are managed, in addition to its operation and practice. The preconditions and essential requirements for developing a CH ecosystem are identified, and a typical case study is selected accordingly. Areas with a complete business ecosystem, including advanced technology and medical services, a payment system, an aging population, geographic isolation, integrated health, and social care, are prevalent. These findings may be beneficial to designing and establishing comprehensive CH implantation and environments. 

To conclude, the evidence and tendency of technological convergence create a demand for innovation and partnering with start-up companies that offer a competitive advantage in innovation. Specifically, it is necessary to innovate both the public and private operation model of the CH ecosystem. This focus will be further explored in future work.

## 2. Method

The method employed in this study is the narrative review. The evolution of names is reviewed from “medical informatics” in the 1970s to “connected health” after 2000, as well as relevant literature of CH, including e-health, telemedicine, telehealth, telecare, and m-health, to discover the trend of technology-related healthcare implementation. This study aims to distinguish CH-related terminology and to identify the trend of CH through narrative review to evaluate its definition, initiation, and evolvement in order to offer management insights and implications. The objective is to understand what is connected and who is cared about in the connected health model so that better applications can be addressed for the benefit of society. The research questions which were taken under consideration are as follows.

1. Why is CH called “connected health”? 2. How is the name of CH evolving? 3. What is issues CH facing? 4. What are the preconditions for applying CH? 5. What are the future trends of CH?

The current status, issues, and market status of CH will be presented, and its future trends will be explored by reviewing how changes in healthcare are managed, in addition to its operation and practice.

**Figure 1:**
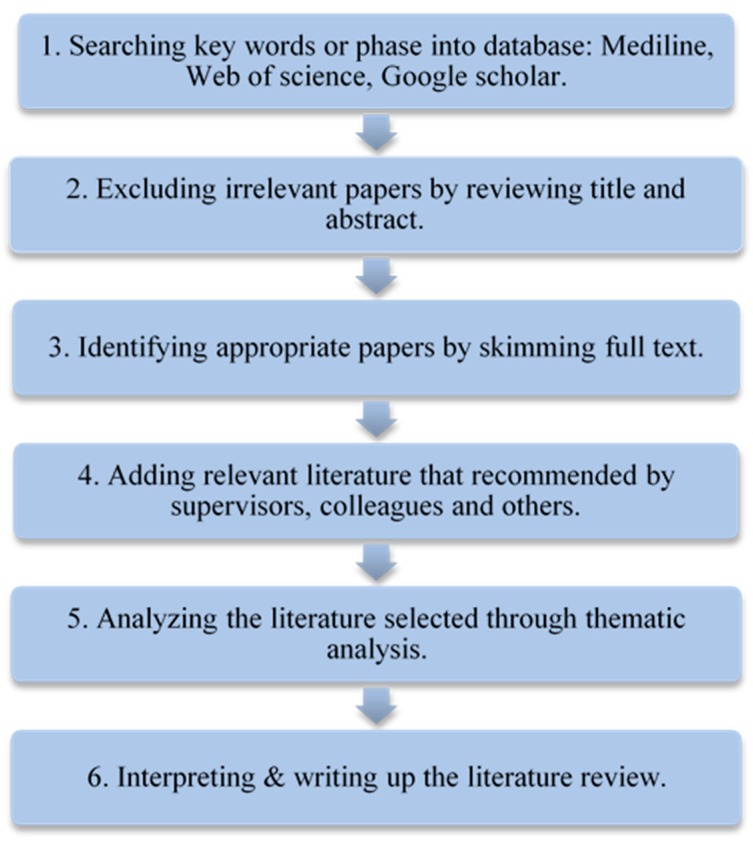
Literature review protocol and process.

**Figure 2:**
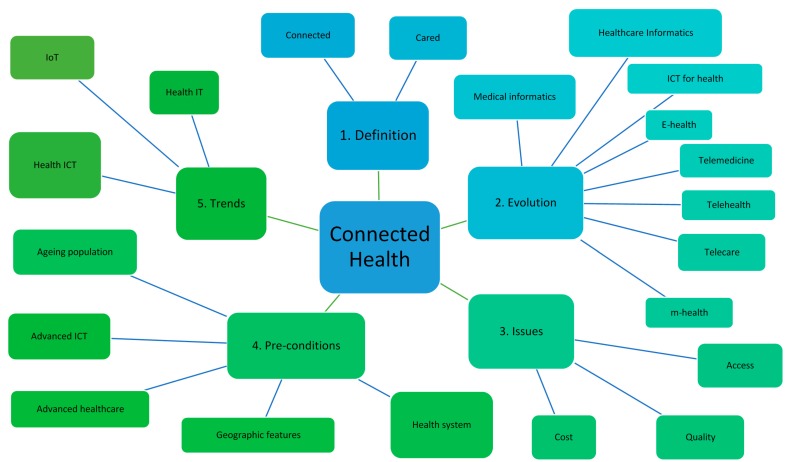
Thematic analysis mapping.

A six-step review protocol is illustrated in Figure 1. A literature search was conducted by using the keywords of “connected health”, “telemedicine”, “telehealth”, “telecare”, and “health innovation” in Medline (including PubMed), Web of Science, and Google scholar, to identify studies of the last fifteen years published in the English language as shown in step 1. Irrelevant papers were excluded by reviewing title and abstract as shown in step 2. A further exclusion was applied to full texts to identify appropriate papers in step 3. Some relevant literature recommended by supervisors, colleagues, and other experts were added to enrich the literature’s database in step 4. From the research, 99 studies were identified that better answer the aim and purposes of the present paper. These studies were evaluated in order to reach a consensus on the eligibility of each one with the proposed research questions. Thematic analysis was employed to analyze the literature selected in step 5. Followed by the findings of the studies’ analysis, this led to the formulation of five thematic categories, namely, (1) Concept Definition and History; (2) The Evolution of the CH concept; (3) Current Issues Facing CH; (4) Pre-conditions and Requirements of CH; and (5) Future Trends of Connected Health as shown in Figure 2. Their outcomes were interpreted in step 6. 

What Figure 2 illustrates is the thematic mapping of literature analysis. It is seen that five themes are identified from CH literature: 1. Definition; 2. Evolution; 3. Issues; 4. Pre-conditions; 5. Trends. Although each theme does not weigh equally, all of them are essential elements that constitute a CH ecosystem. It is noticeable that the evolution of the CH concept plays a relatively important role among them. The perspective of name omen is implicated through the illustration. The themes identified will be further discussed in the following section.

## 3. Results

### 3.1. Definition and History

#### 3.1.1. Definition

Broadly speaking, connected health (CH) can be seen as an umbrella term covering the entire telemedicine family. CH is defined as a model that utilizes technology to maximize healthcare resources and provide increased, flexible opportunities for patients to engage with clinicians and better self-manage their care. In order to achieve these goals, many technologies, such as telemedicine and mobile health, are used to facilitate remote, mobile, and site-to-site medical care [1].

Das and Goswami’s definition (2013) also covers the concept of remote healthcare and the application of an information technology system, which includes a “Telecare medicine information system, personally controlled health records system, and patient monitoring” [14]. They further point out that “in such applications [CH applications], user authentication can ensure the legality of patients”. Apparently, the term CH is seen as a new lexicon for telemedicine, even though it may have a greater focus on the connection methods between clients and healthcare professionals.

Although the role technology plays in CH is significant, it is not just about technologies, but also about connecting people and information within the healthcare system [15]. This definition reveals that technology is relevant and exciting, but it is just a part of the picture, and patient care pathways, business, and revenue models, data analytics, and more should also be included.

It seems to be that CH is not only about technology and people, but also management. According to the definition provided by the literature reviewed, connected health is a new model for health management. It puts the correct information in the right hands at the right time. It allows patients and clinicians to make better decisions: decisions that can save lives, save money, and ensure a better quality of life during and after treatment [16]. Better outcomes can be delivered by a superior allocation of health resources through better management and integration.

There is a tendency for CH to be considered as a combination of people, processes, and technology from a socio-technical perspective, which can be seen in the definition of Caulfield and Donnelly [17]. According to this definition, CH tends to relate to more patient-centered care, as the patient is put at the center of the stakeholders’ connecting process across the spectrum, from the home to the acute care setting. In this case, a more proactive episodic healthcare model can be enabled, compared to the reactive one in conventional healthcare. With the assistance of technology, healthcare professionals, patients, and/or carers may be expected to engage in healthcare in an empowered manner.

#### 3.1.2. History

According to Kvedar, Coye, and Everett’s review, CH’s history can be traced back to the late 1970s in the US and has its root in telemedicine (TM) and telehealth (TH) [1]. These programs are undertaken to improve healthcare access or the shortage of care providers. Compared to TM and TH, CH has a broader concern, related to the cost, quality, and efficiency of healthcare, especially for chronic conditions. Furthermore, it encourages consumerism through patient education and feedback [14,18]. Many efforts have been made to conduct data integration outside the traditional healthcare setting, such as the implementation of electronic patient records. The term telemedicine comprises two parts: “tele” from Greek and “medical” from Latin. When combined, this word means “healing at a distance” [19]. Although telehealth is considered to be an expansion of TM, it has different features from TM. One of its characteristics is the inclusion of the concept of prevention in its practices. TM focuses on perspectives on healing and curing from a distance more than anything else. It was originally employed to support the function of administration and education in TM. Now, it has expanded to various technology solutions and provisions.

#### 3.1.3. Redefining Connected Health

Although the term “connected health” has been increasingly used recently to describe a new technology-enabled model of healthcare delivery, a standard definition for connected health has not been proposed. CH is comprised of two parts: “connected” and “health”. Therefore, the definition should tie up the two concepts of “staying healthy” and “keeping connected”.

According to the World Health Organization (WHO), health includes three aspects of wellbeing: (1) physical, (2) mental, and (3) social [20]. Health includes not only a good physical state but also a good mental and social state [20,21]. Based on WHO’s definition, to achieve “health”, these three aspects need to be connected. In order to achieve this goal, the domains of systems, devices, and people need to be addressed. Possible barriers related to distance, time, workforce, and limited resources need to be managed.

According to Caulfield and Donnelly, all stakeholders in the process, including systems, people, and devices, need to be “connected” [17]. In order to overcome possible barriers of distance and time, a remote service needs to be offered, and self-monitoring needs to be aimed at compensating for the shortage of healthcare providers. Moreover, when it comes to the issue of limited resources, all these connections should aim to maximize resources by integration. The Internet is supposed to be a tool that connects; however, there are still some infrastructure and financial issues to be overcome. How each player in the CH ecosystem can stay connected, stay healthy, and maximize resources must be understood.

### 3.2. Evolution of the Connected Health Concept

#### 3.2.1. Development

According to Topol, preventing chronic illness is the biggest unfulfilled dream in healthcare [20]. Therefore, some practical models, such as TM, TH, telecare (TC), and CH, have emerged with the aim of meeting this goal. Governments and care providers tend to seek out the assistance of Information and Communications Technology (ICT) when faced with a rising demand for healthcare and limits on the capacity of the health system. A possible solution that promises cost-effectiveness and efficiency then needs to be proposed to meet needs [1,22], such as in the case of CH.

Telecare includes general nursing tasks, physical therapy, social work, nutrition and health consultation, meal delivery, patient transportation, and emergency help provision. The service should be a holistic care package that takes into account the life quality of those being cared for. Community and home care are promoted by scholars and governments as the model for aging, as it can satisfy the needs of most care receivers. One of the strengths of TC is supporting the concept of “aging in place” [23,24,25]. Remaining in the home is preferred by many senior citizens, as they would like to postpone or even avoid nursing home care if possible. It is also essential that CH can meet the needs of intensive care units (ICU) across the country, as the supply of intensive care providers is not adequate, especially in small communities and rural hospitals.

Because of the limitations of telemedicine, such as poor information transmission, low user acceptance, and security anxiety, other models are emerging. Connected health has its roots in telemedicine [26]. It was initially used only for serving people in remote areas and has now expanded to cater to those who have chronic conditions [27]. Many pilot schemes have been applied in Veteran Health Affairs [1,28]. According to Das and Goswami, connected health has several applications, including as a telecare medicine information system, a personally controlled health records system, and patient monitoring [14]. In such applications, user authentication can ensure the patient’s right in terms of legality.

Politics always plays a role in healthcare, as political polls and surveys are frequently considered, and consumers, administrators, employers, and clinicians are major concerns [29]. Apart from this, many other factors also increase the need for better healthcare delivery [30]. This demand may relate to a greater speed, efficiency, and cost transparency, as well as vastly improved access to information about companies’ offerings in other industries [31]. Consumers in healthcare are calling for more comprehensive and higher-quality products and services from the healthcare system. Demand is also raised by push power from technology, in the form of the ubiquity of the internet, mobile phones, and electronic devices, which provide inexpensive, but mass-available, offerings to consumers [32]. Therefore, CH experts speculate that changes in consumer behavior will influence the development of healthcare.

The digital revolution has offered healthcare the promise of a more cost-effective and efficient service [33]. Many different terms have been used to describe the intervention of technology in healthcare and sometimes create confusion for readers [19]. Therefore, the authors would like to discuss terminology relevant to connected health to clarify confusion and bring meaning to the topic. A comparison of different terms is undertaken here to determine the domain, evolution, and development of the various concepts.

Today, researchers tend to use the term *connected health* to refer to the inclusion of systems and people in the application of ICT to healthcare [17,19]. However, according to Rossi Mori et al., the name used in every period represents the “destiny” of that time [19]. The story should thus start from the time of medical informatics in 1970 [19,34]. The technology was initially applied to healthcare to differentiate the application fields. As “medical” is used here is an adjective for the noun “informatics”, it suggests that the focus was on “informatics” rather than “medical”. “Medical” here emphasizes the treating or healing of people with problems related to health.

In the 1980s, with the appearance of the term “Healthcare Informatics”, it would seem that the focus shifted to “healthcare” [35,36]. Afterward, with the expansion of the internet and local networking, the function of communication merged with Information Technology (IT). Therefore the term “ICT for health” began to emphasize the importance of communicating function using isolated applications [37,38]. In the meantime, the focus moved from “healthcare” to “health” issues. The “e-” prefix is now applied to a wider range of health topics with an electronic connection and is known as “e-health” [39,40]. Hence, the theme of “health” has eventually become the main concept, and ICT or electronic applications only play a secondary role in this context.

**Figure 3:**
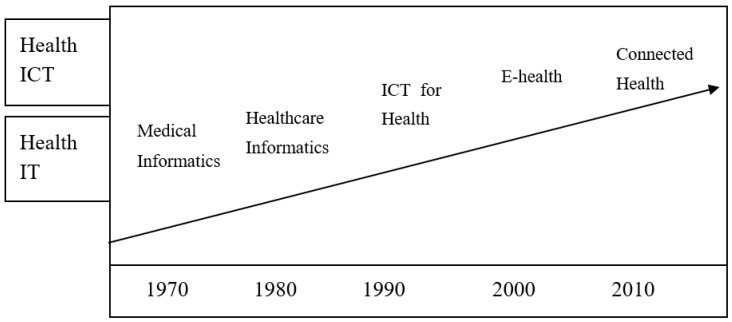
The evolution of names, from “medical informatics” to “connected health” [19].

Connected health was introduced to integrate the different areas and roles in the field (shown in Figure 3) [19]. The focus here is on “health”, and “connected” is an adjective used to describe the concept. ICT should retain an assistive role, and the stress should be on the health of people [41]. This new era of care provision aims to integrate social and healthcare services through the use of ICT.

#### 3.2.2. E-health, M-health, and U-health

**Figure 4:**
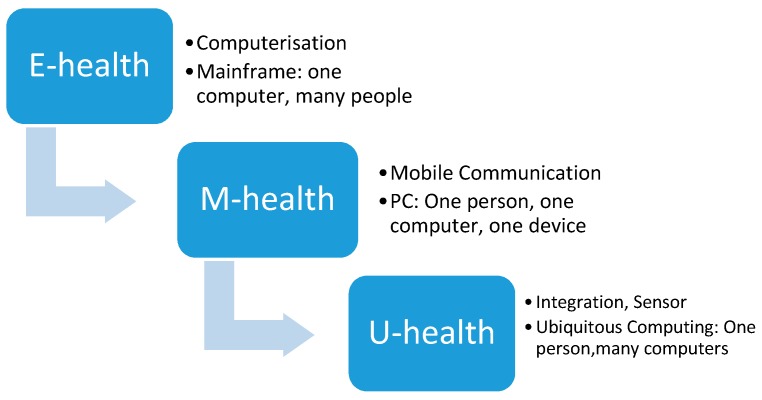
Evaluation of computing (summarized by the authors).

Rossi Mori et al. have provided a clear picture of the development of names and their meanings and the evolution of the focus of the health sector and ICT industry. Although the term e-health is relatively new and all-inclusive, technology continues to progress [19,42]. The terms m-health and even u-health have emerged to respond to the needs of the era [43,44,45,46]. The industrial market for ICT services has matured for wide deployment. Therefore, u-health appears to assist with integration to make health services more accessible [46] (shown in Figure 4).

With the diffusion of mobile phones and tablets, the term m-health has emerged to describe the wireless application of TH services [47]. With the proliferation of mobile devices, the term m-health has been increasing in popularity when it comes to wirelessly accessing services via mobile devices. Moreover, with demands for mobility, u-health has been emerging as a more convenient and efficient healthcare delivery service. From the perspective of technology input in healthcare, some commonly used terms are medical informatics, healthcare informatics, ICT for health, and e-health. They cover similar areas to the ones mentioned above. However, their range can be broader in covering technology practices and the evolving nature and advances of technology. Nevertheless, to include the whole family of technology-based health services, CH may be a very helpful umbrella term. 

#### 3.2.3. Telemedicine, Telehealth, or Telecare

As mentioned above, the term used to describe the use of technology to deliver healthcare services in a remote context was originally telemedicine (TM). It referred to the Latin and Greek words for “medical” and “tele”, which combine to mean “healing at a distance” [46]. From the definition listed above, it is clear that TM’s attention to healing and treating has gradually shifted to education and disease prevention. The rise of TM suggests that the prevention of disease is becoming more important and that educating patients on self-management may be more cost-effective. Therefore, TH has retained the TM functions of diagnosing and treating remotely and has continued to develop to allow patients to self-manage and monitor, for the purpose of education and prevention [44]. This demonstrates the shifting of the emphasis of medicine to health, from TM to TH. Due to the complexity of healthcare, many different terms emphasize the focus of similar models in different features [45]. However, following technological developments, many more functions have been inputted to TM. TH and TC may be some of the most frequently mentioned terms and are relatively similar to each other [45,46,47]. Therefore, some prefer to use the term “tele-healthcare” to cover them both.

TH usually refers to the monitoring of those who have been diagnosed with specific conditions [48]. It includes alarm systems that can help patients access health services in the golden period, which is key to saving their lives. It aims to treat patients in the early stage to achieve more effective results [46,48]. TC is more accessible than TH because it does not require specialists or specialist nurses, but instead, social workers or anyone who has been trained to conduct the service. TH, on the other hand, is led by health professionals, such as GPs or specialists, due to the requirements of medical law and concerns about health conflicts. Many pilot schemes have been narrowed down to TC or TH because the coverage of CH is too broad to be focused. Therefore, the terms TC and TH can be used interchangeably to discuss relevant practices [48,49].

### 3.3. Current Issues Facing of Connected Health

The current status and issues facing CH were divided into the subsections of accessibility, quality, and cost. Countries all over the world are keen to implement CH [49]. However, the importance of offering evidence to support its feasibility and effectiveness is greater than considerations of who the players may be [50,51]. The outcomes and evidence of CH to date will be reviewed based on the two dominant markets: the US and the EU [52,53]. Despite CH evaluation criteria and categories being variable in existing literature, most studies seek to solve the three key challenges of accessibility, quality, and cost in healthcare.

#### 3.3.1. Accessibility

Access to specialists refers to an approach employed to extend services and specialized knowledge across geographic boundaries [54,55,56]. This was once a dream; however, with the help of digital imaging innovation and the Internet, the extension of services has become feasible through interactive video conferencing and video recording [57]. Specialty physicians are now able to provide services in a time- and place-independent manner. Video conferencing is low in cost and provides benefits to patients, especially when they are living far from healthcare providers [58]. Video recording is called a “store and forward” strategy and is more widely adopted by taking advantage of high-resolution cameras in smart devices in order to make healthcare services more efficient [1]. A dermatologist at Kaiser Permanent in San Diego, California, treats approximately 800 such cases per month using this method, handling 50 percent more cases than would be possible in face-to-face visits [1,2]. Although more and more patients have begun using online services through smartphones, the outcomes have not yet been proven to be effective [57]. Moreover, those who are only comfortable with diagnosing conditions based on directly observing the patient have been slow to adopt these technologies [48,49,50].

#### 3.3.2. Quality

The quality of health outcomes can be measured by reductions in the hospital readmission rate and chronically ill patients’ mortality rate, as well as increasing the care efficiency. Remote monitoring has contributed to reducing the all-cause mortality rate by 20%, and the hospital readmission rate for chronic heart failure by 21%, based on a sample of over 4000 patients in 14 randomized controlled trials [59]. The results of the UK’s Whole System Demonstrator (WSD) also shows a 45% reduction in mortality rates among 3153 subjects [49,60]. An e-referral service model is broadly employed in the US to allow primary care providers to exchange privacy-protected, template email messages with specialists. Programs of this kind have been developed at health institutions such as San Francisco General Hospital, the Mayo Clinic, and UCLA [1]. Each implementation has produced shorter wait times, improved the preparation of patients for specialty visits when required, and strengthened primary-care-provider–specialist collaboration and satisfaction [61]. Evidence reveals that the number of in-person specialty visits can be reduced by 29% or more in this way [1]. However, the quality of healthcare is not defined by better and more comfortable services, but also by whether or not it allows patients to keep their freedom and dignity and to maintain independence [62]. The Veterans Health Program in the US is able to facilitate the independent living of 36% more patients than long-term residential care [63]. In the UK, millions of people benefit from TC services, which provide person-centered technologies to support individuals, such as those with dementia or those at risk of falling, helping them to maintain independent living [64].

#### 3.3.3. Cost

Cost-saving and cost-effectiveness can be measured by the reduction of major diseases in healthcare. Chronic heart failure and ICUs are two typical cases [65]. The former is a major consumer of health resources, while the latter accounts for approximately 1% of GDP spending annually in the US. Although chronic heart failure is a commonly diagnosed condition, its prognosis is still poor in prevention. According to a review of clinical trials, 30 randomized trials for chronic heart failure have been established. These multidisciplinary, non-pharmacological approaches have shown their effectiveness in improving the outcomes of this chronic condition [66]. More than 3000 chronic heart failure patients received care via in-home monitoring with this approach, with three to four nurses caring for a daily panel of 250 patients. The program generated cost savings of more than $10 million over a six-year period. Telemonitoring shows benefits in extending services to larger populations of patients. In the US, ICUs are offering care to six million patients per year, at an annual cost of over $100 billion [1]. The use of tele-ICU technologies allows caregivers to leverage coverage over more ICU beds and increase productivity. This can be done by providing direct consultation and management of ICU patients from a distant site through remote two-way audio, visual, and physiological monitoring [67,68]. Through remote monitoring and videoconferencing, the four-year Veterans Health Program has reached up to 120,000 veterans over four years and generated annual savings of $1999 per patient [1]. Using similar practices, there has been a 20% reduction in emergency admissions and an 8% reduction in tariff costs in the WSD, involving over 6000 patients across Newham, Kent, and Cornwell [49,60]. These results positively contribute to savings in health resources and cost-effectiveness in the cases of chronic heart failure and ICUs; however, the outcomes for other practices or remote areas may not show a significant contribution to cost-effectiveness.

## 4. Discussion

### 4.1. Connected Health in Operation and Practice

CH emerges from the background mentioned above. It involves healthcare, social care, and wellness, all of which overlap due to the complex nature of healthcare [17,52,53]. TC is more relevant to social care, while TH is more concerned with healthcare. They are both included in the domain of CH as they both promote the goal of wellness. The government, industry, and academia all perform an equally important role in enabling CH, forming a triple helix partnership [69,70,71]. These three main roles are only general terms in the CH ecosystem. Specifically, it is necessary to consider the collaboration of significant players from different disciplines in CH practices, such as software developers, hardware manufacturers, healthcare clinical and care service providers, support service providers, total solution companies, and end-users (patients or clinicians) (as shown in Table 1). 

Although CH covers various practices, it can be classified as two main platforms: self-management (patient empowerment) and remote-care (remote connectivity) [72,73]. The former includes all the practices that enable patients to be empowered to manage their own health [74]. The latter refers to platforms and applications that facilitate remote patient care [75]. In practice, CH is usually corporately operated by three types of care models, including home care, community care, and institutional care, to fulfill the different needs and locations of the target segment [54]. With the promotion of “aging in place”, home and community care models are becoming the focus of healthcare as they are helpful in increasing the quality and cost-effectiveness of care compared to the institutional care model [38,76,77]. Geographically, western countries tend to be more developed in institutional care, while Asian countries stress home and community care because of cultural differences.

CH has two main markets—the United States and the European Union—where there is relatively advanced medicine and broad internet coverage [78]. CH is especially needed in rural and remote areas where there is less healthcare available, and where isolated communities may be subject to severe weather conditions, such as in Scotland, Finland, Ireland, Northern Ireland, Norway, and Sweden [79]. Isolation increases the need for being connected, which echoes the principle of collaboration in CH. It is not surprising that although countries such as Australia already have CH practices, they are still willing to collaborate with the US and Europe as part of a multidisciplinary team [80]. Recently, Asian countries have also taken an active role in the CH market due to demographic challenges, such as an aging population and a low birth rate. With the help of advanced technologies, both in medicine and information communication, cases such as Japan, Singapore, Korea, and Taiwan area have achieved significant results in CH practices [81,82].

Compared to the EU, the US tends to be more developed and mature in its CH development. Many EU programs are still in the trial and pilot stage, while the US has many cases of commercialization [81]. This may be influenced by the respective national health systems, as healthcare in the US tends to be privatized, while that in the EU is relatively publicly funded [83]. Many CH practices can be hospital-led rather than government-led, and hospitals can experience commercial pressures. In this case, they are keen to develop a relatively sustainable business model. On the contrary, EU countries are mainly social-welfare oriented and rely on government funding to initiate or sustain CH implementation. Therefore, their focus is more on testing the feasibility of CH practices and cost-saving rather than developing a business model for earning profits. Their coverage areas are not limited to smaller and fewer areas, unlike government funding. The selection of pilot areas for trials is significant. If they are not appropriate areas for CH, the validity and credibility of the results will be a concern. For example, remote areas are appropriate for CH research according to the literature; however, the results of pilot studies are not clearly represented by the samples in the trials.

In addition, even though the outcomes of CH focus on a reduction of the mortality rate, readmission rate, and medication adherence rate, there are still many other ways to measure healthcare outcomes [67]. This may suggest that CH’s performance is not significant beyond these measurements. Although CH demonstrates positive outcomes in accessibility, quality improvement, and cost savings, it has mostly been limited to small-scale trials to date. The largest scale is the nationwide Veterans Health Program in the US. However, this is limited to a specific category and may not be able to represent the total population. For example, the issues faced by ethnic minorities are less studied in US cases, but this does not mean that they are not important. Other cases that have significant outcomes of performance are narrowed down to certain specialties, such as heart failure, diabetes, and ICU [68]. A broader range of CH practices still remains to be explored.

### 4.2. Pre-conditions and Requirements of Connected Health

Findings of this study suggest that there are certain pre-conditions and essential requirements that can contribute to the implementation of comprehensive CH services, despite challenges remain. CH is suitable for development in areas where a complete business ecosystem has advanced software development, such as in medical device companies [84,85,86]. Apart from these, other advantageous pre-conditions can be identified, such as a single-payer system, integrated health and social care, and a population where many chronic conditions are prevalent [22,86,87]. An insurance payer system is important to CH business sustainability in deciding whether governments or companies need to bear the enormous cost of insuring their citizens or employees [88]. CH overlaps the sectors of health and social care [89]. Therefore, integrating both into one department is helpful for CH implementation. In addition, a population with many prevalent chronic conditions is essential for practicing CH, as there is a stronger need for its services compared to a healthier population [90,91]. Moreover, one of the features of CH is to extend its service to a broader population in distant or remote areas [72,92]. Therefore, it is important that CH is proven to be feasible in remote areas. It would be desirable to research CH in an area where these conditions and requirements exist in order to improve the validity and credibility of results.

Both the US and European countries have advanced technology and medicine, but they are very different in terms of their healthcare systems [93]. In addition, their differences place them at opposite ends of the range. Healthcare in the US tends to be privatized, while it is relatively socialized in Europe [53]. The former seems to focus more on commercialization, while the latter stresses social welfare. Both US and EU markets are representable in their field, but they may not be typical of universal CH. It may require collaborations in knowledge management and knowledge transformation from different countries to identify a typical case study that can fulfill CH requirements and cover these differences between countries, as this would be more likely to be beneficial in offering solutions to CH issues [72]. A typical case study is the one that meets all the CH pre-conditions and essential requirements. Not only is there advanced technology and medicine, but the ecosystem for boosting CH is also complete and comprehensive. Regions such as Taiwan and Northern Ireland, which have recently integrated health and social care, are ideal cases to be researched. Their chronic conditions are prevalent due to an aging demographic. Geographically, they have populations in urban areas, remote areas, and isolated islands. Moreover, a mixture of public and private healthcare systems is also beneficial. These features suggest ideal selection criteria to study CH.

### 4.3. Future Trends of Connected Health

#### 4.3.1. From Health Information Technology to Health Information Communication Technology

There is a tendency to express the concept of “medical informatics” through the term “ICT for healthcare”. This suggests that the role of communication in IT is becoming significant. IT is no longer an isolated device, but something that can help in communicating with each other. It is essential to the health sector as it implies that medical devices should be more integrated than isolated. The focus was on technological devices during the time of “medical informatics” [34,36,38,54]. However, it has moved to a system connecting people during the era of “connected health”. The evolution of e-health into m-health and u-health shows the tendency to use wireless applications to make service delivery more available and easily accessible [94].

From the perspective of “from medical informatics to connected health”, the concepts of health and disease prevention have already been included in health concerns when it comes to achieving the goal of wellness [79]. Even though the focus was on care in the time of “healthcare informatics”, it is not the ultimate goal of this evolution. Therefore, a tendency to move from medicine to wellness can be seen from this transformation. The emphasis was on informatics during the time of “medical informatics”; however, it shifted to health during the time of “ICT for health”, and has maintained this focus in the time of “e-health” and “connected health”. This implies that technology can contribute to the area of health.

In terms of the care model, there is a tendency to reduce institutional care and shift to home or community care for both US and EU markets. Nevertheless, some Asian countries have also achieved distinguished outcomes in this century. Developed countries, such as the US and Canada, also learn from Asia. Recently, Asian countries have been keenly developing CH-associated practices due to advancements in technology, social-economic growth, and an aging population. Although many of these CH practices are based on learning from the US and Europe, some pilot schemes have shown significant results.

#### 4.3.2. Future: The Internet of Things

Advances in telecommunication and technology will significantly influence the practices of CH [49] as technology plays the role of an enabler rather than the solution. According to a previous review, a new data-driven revolution will be applied to the development of technology in healthcare. A network of linked devices and objects that are collecting, sending, and receiving data about people, environments, and processes, without human interactions or inputs, will enable a new model of “connected health”, not only for the chronically ill but for the entire population [93]. The focus will be shifted from the performance of a diagnosis or treating a patient in a clinical environment with a device, to data-driven solutions. The White Paper predicts that the future trend of Internet technology will be a move from a fixed Internet in the 1990s, to a mobile internet in the 2000s, and to “things connected to the internet” by 2020 [32,92].

**Figure 5:**
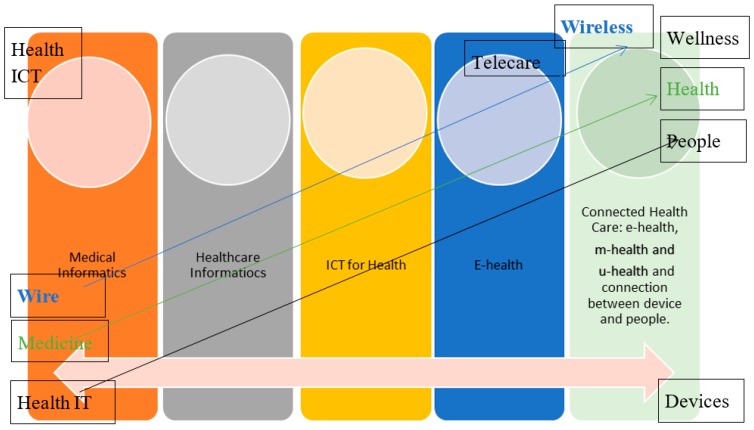
The development of Information and Communications Technology (ICT) in health.

Based on the literature reviewed above, a new feature of technology evolution in health can be introduced, as shown in Figure 5. This figure shows three shifting directions in technological evolution: (1) from IT to ICT, (2) from devices to people, and (3) from wired to wireless. It also shows the shifting directions of health evolution: from medicine to wellness and from technology-centered to health-centered. After reviewing the evolution of technology in health, a more comprehensive understanding can be presented, as shown in Figure 5. When it comes to practice, there are still some different terms which emphasize practice in different contexts. This study suggests that the tendency of a more connected system will collaborate with healthcare.

## 5. Conclusions

This study introduces the emergence of CH by reviewing the evolution of CH, including its definition, history, evolution, and development, which are presented alongside issues to be overcome. It contributes to the knowledge through providing management insights and implications through the name omen perspective of reviewing relevant literature. Pre-conditions and essential requirements for boosting the CH ecosystem are identified, and evidence suggests that CH in Taiwan is a suitable area for research into CH. This review also discusses whether CH is a buzzword or a practice that can contribute to an aging society. These range from telehealth services to ambient assisted living research programs and the use of new ICT solutions in diverse aspects of acute and chronic clinical care. Future trends of CH are proposed for managers for implementations. Specifically, it is necessary to innovate both the public and private operation model of the CH ecosystem. This focus will be further explored in future work.

## Figures and Tables

**Table 1:** Roles in connected health (CH) ecosystem (Source: [17] Edited by the author, 2020).

Roles	Ecosystem		Target Segment
Government/Academia/Industry	Software developers	N/A	End users
	Hardware manufacturers	N/A	
	Healthcare	Clinical and care service providers	
	Support service providers	Internet and telecom companies	
	Total solution companies	Combination of these companies	
